# Inhibitory Effect of Biotransformed-Fucoidan on the Differentiation of Osteoclasts Induced by Receptor for Activation of Nuclear Factor-κB Ligand

**DOI:** 10.4014/jmb.2203.03001

**Published:** 2022-07-08

**Authors:** Bobae Park, Sun Nyoung Yu, Sang-Hun Kim, Junwon Lee, Sung Jong Choi, Jeong Hyun Chang, Eun Ju Yang, Kwang-Youn Kim, Soon-Cheol Ahn

**Affiliations:** 1 Department of Microbiology & Immunology, Pusan National University School of Medicine, Yangsan 50611, Republic of Korea; 2 Section of Pulmonary, Critical Care and Sleep Medicine, Department of Internal Medicine, Yale University School of Medicine, New Haven, CT 065510, USA; 3 Department of Biomedicinal Science and Biotechnology, Pai Chai University, Daejeon 35345, Republic of Korea; 4 Spine Center, Bone Barun Hospital, Yangsan 50612, Republic of Korea; 5 Department of Clinical Laboratory Science, Daegu Haany University, Gyeongsan 38610, Republic of Korea; 6 Korean Medicine Application Center, Korea Institute of Oriental Medicine, Daegu 41062, Republic of Korea; 7 Department of Molecular Medicine, University of Texas Health at San Antonio, San Antonio, TX 78229, USA

**Keywords:** Biotransformation, fucoidan, osteoclast differentiation, receptor for activation of nuclear factor-κB ligand, tartrate-resistant acid phosphatase

## Abstract

Bone homeostasis is regulated by constant remodeling through osteogenesis by osteoblasts and osteolysis by osteoclasts and osteoporosis can be provoked when this balance is broken. Present pharmaceutical treatments for osteoporosis have harmful side effects and thus, our goal was to develop therapeutics from intrisincally safe natural products. Fucoidan is a polysaccharide extracted from many species of brown seaweed, with valuable pharmaceutical activities. To intensify the effect of fucoidan on bone homeostasis, we hydrolyzed fucoidan using AMG, Pectinex and Viscozyme. Of these, fucoidan biotransformed by Pectinex (Fu/Pec) powerfully inhibited the induction of tartrate-resistant acid phosphatase (TRAP) activity in osteoclasts differentiated from bone marrow macrophages (BMMs) by the receptor for activation of nuclear factor-κB ligand (RANKL). To investigate potential of lower molecular weight fucoidan it was separated into >300 kDa, 50-300 kDa, and <50 kDa Fu/Pec fractions by ultrafiltration system. The effects of these fractions on TRAP and alkaline phosphatase (ALP) activities were then examined in differentiated osteoclasts and MC3T3-E1 osteoblasts, respectively. Interestingly, 50-300 kDa Fu/Pec suppressed RANKL-induced osteoclasts differentiation from BMMs but did not synergistically enhance osteoblasts differentiation induced by osteogenic agents. In addition, this fraction inhibited the expressions of NFATc1, TRAP, OSCAR, and RANK, which are all key transcriptional factors involved in osteoclast differentiation, and those of Src, c-Fos and Mitf, as determined by RT-PCR. In conclusion, enzymatically low-molecularized 50-300 kDa Fu/Pec suppressed TRAP by downregulating RANKL-related signaling, contributing to the inhibition of osteoclasts differentiation, and represented a potential means of inducing bone remodeling in the background of osteoporosis.

## Introduction

Bone is one of the central skeletal systems in our bodies and it supports all shapes and sizes of the body, protects vital organs and allows movement with skeletal muscles [1]. Also, it acts as a minerals reservoir from the body’s supply, especially calcium, which is essential for homeostasis of the nervous and muscle systems [1]. It is composed of the bone cells, such as osteoclasts and osteoblasts, and the bone matrix comprised of collagenous crystal and mineral ingredients. Its normal structure is consistently maintained by the bone homeostasis between osteogenesis and osteolysis. Thus, the differentiation to osteoblasts and osteoclasts is very important to regulate bone formation and resorption. Bone undergoes constant turnover to ensure the maintenance of healthy bone structure through various phases of the bone remodeling cycle. These phases can be largely divided into activation, resorption, reversal, formation, and quiescence or resting phase [2, 3].

Osteoclasts are derived from the hematopoietic lineage and are specifically responsible for bone resorption. They attach to the surfaces of aged bones and dissolve bone matrix by releasing particular catabolic enzymes and acids, representing their specific feature to break down bone. Macrophage colony-stimulating factor (M-CSF) and receptor for activation of nuclear factor-κB ligand (RANKL) are essentially required to stimulate osteoclastogenesis [4, 5]. Osteoclasts express specific differentiation factors, such as microphthalmia-associated transcription factor (Mitf), Spi-1 proto-oncogene (SPI1, also known as PU.1), Src, c-Fos, and nuclear factor of activated T-cells c1 (NFATc1), which act as signaling proteins at each differentiation stage. The induction of these transcriptional activities leads to up-regulation of tartrate-resistant acid phosphatase (TRAP) and osteoclast-associated receptor (OSCAR) [4,6]. On the other hand, osteoblasts are derived from mesenchymal stem cells (MSCs) and are responsible for synthesis of bone matrix and renovation of older bone, which is influenced by activated vitamins, hormones and growth factors. The differentiation of osteoblasts is sequentially mediated by the activation of bone morphogenetic protein (BMP) and Cbfa1/Runx2, specific markers of osteoblasts [7, 8]. Runx2 is considered the major contributor to osteoblasts proliferation and differentiation during bone remodeling. In addition, osteopontin, bone sialoprotein, osteocalcin and alkaline phosphatase (ALP) are essential modulators of osteoblasts maturation under the regulatory control of Runx2 [9]. Interestingly, RANKL and osteoprotegerin secreted from the osteoblasts also induce transcription factors required for osteoclasts differentiation.

Osteoporosis is a typical bone disease caused by reduced estrogen leves, physical stress and imbalanced synthesis between osteoclasts and osteoblasts. Treatments include bisphosphonates, hormone-related therapy, and bone-building medications. Bisphosphonates system are the most widely used but gradually suppress osteogenesis after administration for several months [10]. Hormone-related therapy right away after menopause helps sustaining bone mineral density but its long-term administration eventually may lead to breast cancer and hypertension [11]. Furthermore, these treatments are inadequate in terms of increasing osteogenesis and preventing bone fractures. To develop therapeutic agents that overcome these side effects, novel-drug strategies based on the use of natural products offer a possible solution because they have fewer side effects and safer long-term administration, and several have been recently reported to modulate osteolysis, osteogenesis, and bone remodeling [12-14]. In particular, fucoidan has been reported to prevent osteoclastogenesis and promote osteoblasts differentiation [15-20]. Fucoidan is a sort of sulfated high molecular polysaccharide and a component of cell wall in the brown seaweed *Undaria pinnatifida*, which is widely cultivated in Korea. Its main component is sulfate fucose though it also contains small quantities of galactose, xylose and glucuronic acid, exhibiting some difference of its constituents depending on its origins [21, 22]. Many authors have reported on the classical activities of anti-cancer, anti-oxidant, anti-coagulant, anti-thrombotic, anti-viral, and immunomodulatory effects [23-27]. And also it has been reported to inhibit tumor angiogenesis, ameliorate inflammatory bowel disease and metabolic syndrome, and improve bowel health [28-30]. Recently studies have shown that low molecular weight of fucoidan inhibits hepatocarcinogenesis, exhibits potential anti-oxidant, anti-coagulant and anti-thrombotic activities, protects endothelial function, and ameliorates non-alcoholic fatty liver disease and basal hypertension in diabetic rats [17, 31-34].

This study was undertaken to determine how to prepare a form of fucoidan that potently redresses the balance between osteogenesis and osteolysis. To achieve this, we treated fucoidan with polysaccharide-degrading enzymes, isolated a low-molecular form of fucoidan by ultrafiltration system, and evaluated its effects on enzymes known to be associated with osteoporosis.

## Materials and Methods

### Reagents

The fucoidan from sea mustard (*Undaria pinnatifida*) was obtained from Bion F&B (Korea), which was estimated as 31-34% sulfate contents according to a certificate of chemical analysis provided by the company. Three different commercial carbohydrate-hydrolyzing enzymes, AMG (rich in amylase), Pectinex (rich in pectinase), and Viscozyme (rich in cellulase, β-glucosidase, xylanase), were purchased from Novozymes (Bagsvaerd, Denmark) for enzymatic biotransformation of fucoidan. Macrophage colony-stimulating factor (M-CSF) and receptor activator of nuclear factor kappa-Β ligand (RANKL) for the differentiation of osteoclast were purchased from PeproTech (UK). α-Minimal essential medium (MEM) containing 100 μg/ml streptomycin, 100 U/ml penicillin, and fetal bovine serum (FBS) were purchased from Gibco (USA). The ascorbic acid and β-glycerophosphate used for inducing the osteoblast differentiation and tartrate-resistant acid phosphatase (TRAP) staining kit for detecting the osteoclast differentiation were purchased from Sigma Chemicals (USA). 3-(4,5-Dimethyl-thiazol-2-yl)-2,5-diphenyltetrazoliumbromide (MTT) was purchased from Duchefa (Netherland). To isolate and purify total RNA, TRIzol reagent was used (Life Technologies, USA).

### Biotransformation and Fractionation of Fucoidan

The enzyme-aided biotransformation of fucoidan was performed as previously reported [42]. Three commercially available carbohydrate-degrading enzymes, AMG, Pectinex, and Viscozyme were used to obtain lower molecular weight of fucoidan. 0.5% (v/v) of fucoidan was resuspended in distilled water, treated with 5% (v/v) of each enzyme, and incubated for 2 days at 55°C. Finally, reactions were then stopped by heating for 10 min at 100°C and clarified by centrifugation at 5,000 *×*g for 20 min at 4°C. To prepare the low-molecular fraction of biotransformed-fucoidan by Pectinex, QuixStand BenchTop system (GE Healthcare, USA) equipped an ultrafiltration membrane cartridge was used, which was composed of pore sizes from 50 to 300 kDa molecular weight cut-off (MWCO) at osmotic pressure at 150-115 rpm. The surface area of the membrane cartridge was 110 cm^2^, equivalent to the application of 1,000 ml sample.

### Differentiation of Bone Marrow Cells to Osteoclast Cells

Primary mouse bone marrow cells were obtained from the femurs and tibiae of a 6-week-old male ICR mouse as described by Lee *et al*. with slight modification [43]. Briefly, hind leg tibiae of a 6-week-old ICR male mouse were aseptically isolated and, after removing cartilage, the bone marrow cells were obtained by flushing with ice-cold HBSS using a syringe. After centrifugation at 1,500 *×*g for 5 min, cell pellets were collected and incubated in α-MEM complete media supplemented with 10% FBS, 100 μg/ml streptomycin, and 100 U/ml penicillin in a 100 mm dish in the presence of M-CSF (30 ng/ml) for 2 days. Adherent cells were deemed bone marrow macrophages (BMMs). To induce osteoclasts differentiation from BMMs, BMMs (2 × 10^4^ cells/well) were then cultured in α-MEM complete medium containing 100 ng/ml of RANKL and 30 ng/ml of M-CSF for 3 days in a 96-well plate with or without biotransformed- fucoidans or ultrafiltrated-fucoidan fractions. All animal experiments were approved by the Ethics Committee of Pusan National University (PNU-2021-2985, May 14^th^, 2021).

### Tartrate-Resistant Acid Phosphatase (TRAP) Activity and TRAP-Positive Multinucleated Cells

To measure TRAP activity using a colorimetric assay, freshly differentiated osteoclasts were harvested, washed with 1x phosphate-buffered saline (PBS), and lysed with 100 μl of lysis buffer (0.5 M Tris, pH 9.0, 150 mM NaCl, 1% Triton X-100) for 1 h at 4°C. The cell lysate was then incubated with 100 μl of phosphatase substrate solution (5 mM p-nitrophenyl phosphate disodium salt, 10 mM sodium tartrate, 50 mM citrate buffer, pH 5.8) for 30 min at 37°C. To verify TRAP activity, 50 μl of supernatant was transferred to new 96-well plate, an equal volume of 0.1 N NaOH was added to stop the reaction, and optical density was measured at 405 nm using a SpectraMax M2e microplate reader (Molecular Devices, Canada). All data were expressed as percentages of non-treated group. In addition, to visualize TRAP activity through visualized images, we used a commercial TRAP staining kit (Sigma Chemicals) according to the manufacturer’s instructions. Briefly, cells were fixed in 10% formalin for 10 min, permeabilized with 0.1% Triton X-100, stained with TRAP staining solution for 1 h at 37°C, washed with pre-warmed distilled water, and air-dried. TRAP-positive multinucleated cells, which should be more than 3 of the number of nuclei, were observed and photographed under a Leica DM inverted microscope system (Germany).

### Osteoblast Cells Culture and Alkaline Phosphatase (ALP) Activity

MC3T3-E1 osteoblastic cells were purchased from the American Type Culture Collection (USA) and cultured in α-MEM containing 10% FBS, 100 μg/ml streptomycin, and 100 U/ml penicillin on the polystyrene-coated dishes. Cell densities were maintained so as not to exceed 70-80% confluency. Briefly, MC3T3-E1 cells were seeded into a 96-well plate at a density of 1 × 10^4^ cells/well and cultures for 24 h. And α-MEM-based medium was replaced with fresh differentiation medium containing 10% FBS, 1% penicillin-streptomycin, 10 mM β-glycerophosphate and 100 mg/ml ascorbic acid as osteogenic agents. And the cells were treated with ultrafiltrated-fucoidan fractions at different concentrations and incubated for an additional 7 days. To determine ALP activity using a colorimetric assay, cells were washed with 1x PBS and lysed by incubation in 100 μl of lysis buffer (0.5 M Tris, pH 9.0, 150 mM NaCl, 1% Triton X-100) for 1 h at 4°C. Cell lysates were then treated with 100 μl of 5 mM *p*-nitrophenyl phosphate solution for 30 min at 37°C and 50 μl of resulting supernatants were transferred to new 96-well plates. The reaction was stopped by adding an equal volume of 0.1 N NaOH and absorbances were measured at 405 nm using a SpectraMax M2e microplate reader (Molecular Devices). All data were expressed as percentages of non-treated group.

### Cell Cytotoxicity

Cytotoxicities were determined using an MTT assay. Briefly, the cells were seeded at 2 × 10^4^ cells/200 μl of differentiated osteoclast or 5 × 10^4^ cells/200 μl of osteoblast MC3T3-E1 per well in a 96-well plate and incubated in α–MEM medium for 24 h. Cells were replaced with 200 μl of fresh α–MEM media, treated with or without different concentrations of biotransformed- fucoidans or ultrafiltrated-fucoidan fractions, and incubated for further 48 h. Ten microliters of MTT solution (5 mg/ml) was then added to each well and incubated for 5 h. The supernatants were removed and 100 μl of DMSO was added to each well. Optical densities were measured at 550 nm using a SpectraMax M2e microplate reader (Molecular Devices). Cell viabilities were expressed as percentages of non-treated group. All data were presented as the mean value±SEM of optical densities in three independent experiments.

### RNA Isolation and Semi-quantitative Reverse Transcription-Polymerase Chain Reaction (RT-PCR)

Total RNA was isolated using TRIzol method and quantified by measuring optical densities at 260 nm and 280 nm using a BioPhotometer (Germany). cDNA was synthesized using Superscript II reverse transcriptase (Invitrogen, USA) with 2 μg of total RNA. The primers used for the PCR reaction were as follows: RANK, 5’-TAC TAC AGG AAG GGA GGG AAA G-3’ and 5’-CCT GCT GGA TTA GGA GCA GTG-3’; NFATc1, 5’-CTC GAA AGA CAG CAC TGG AGC AT-3’and 5’-CGG CTG CCT TCC GTC TCA TAG-3’; OSCAR, 5’-CTG CTG GTA ACG GAT CAG CTC CCC AGA-3’and 5’-CCA AGG AGC CAG AAC CTT CGA AAC T-3’; TRAP, 5’-CAG TTG GCA GCA GCC AAG GAG GAC-3’and 5’-GTC CCT CAG GAG TCT AGG TAT CAC-3’; Src, 5'-AGC AAC AAG AGC AAG CCC AAG GAC-3' and 5'-GTG ACG GTG TCC GAG GAG TTG AAG-3'; c-Fos, 5'-ACT TCT TGT TTC CGG C-3' and 5'-AGC TTC AGG GTA GGT G-3'; Mitf, 5'-GAC ATC ATC AGC CTG GAA TC-3' and 5'-ACA GAG GCC TTG AGA ATG GT-3'; PU.1, 5'-CGC AGC TAC AGC AGC TCT AT-3' and 5'-GAA CTG GTACAG GCG AAT CT-3'; HPRT, 5’-GTA ATG ATC AGT CAA CGG GGG AC-3’and 5’-CCA GCA AGC TTG CAA CCT TAA CCA-3’. PCR reactions were incubated for 5 min at 95°C and the amplification was performed for 30 sec at denaturing temperature of 95°C, for 30 sec at annealing temperature of each primer, and finally for 30 sec at extending temperature of 72°C. All bands were quantified using Image J software (NCB/NIH, USA).

### Statistical Analysis

All results were presented as the mean ± SEM of at least three or more experiments. The significances of differences between mean values were analyzed by ANOVA test (Tukey-Kramer multiple comparisons). Statistical significance was accepted for a value of **p < 0.01 and ***p < 0.001 as indicated.

## Results

### Effects of Biotransformed-Fucoidans on Cell Viability and Osteoclasts Differentiation

**Fig. 1:**
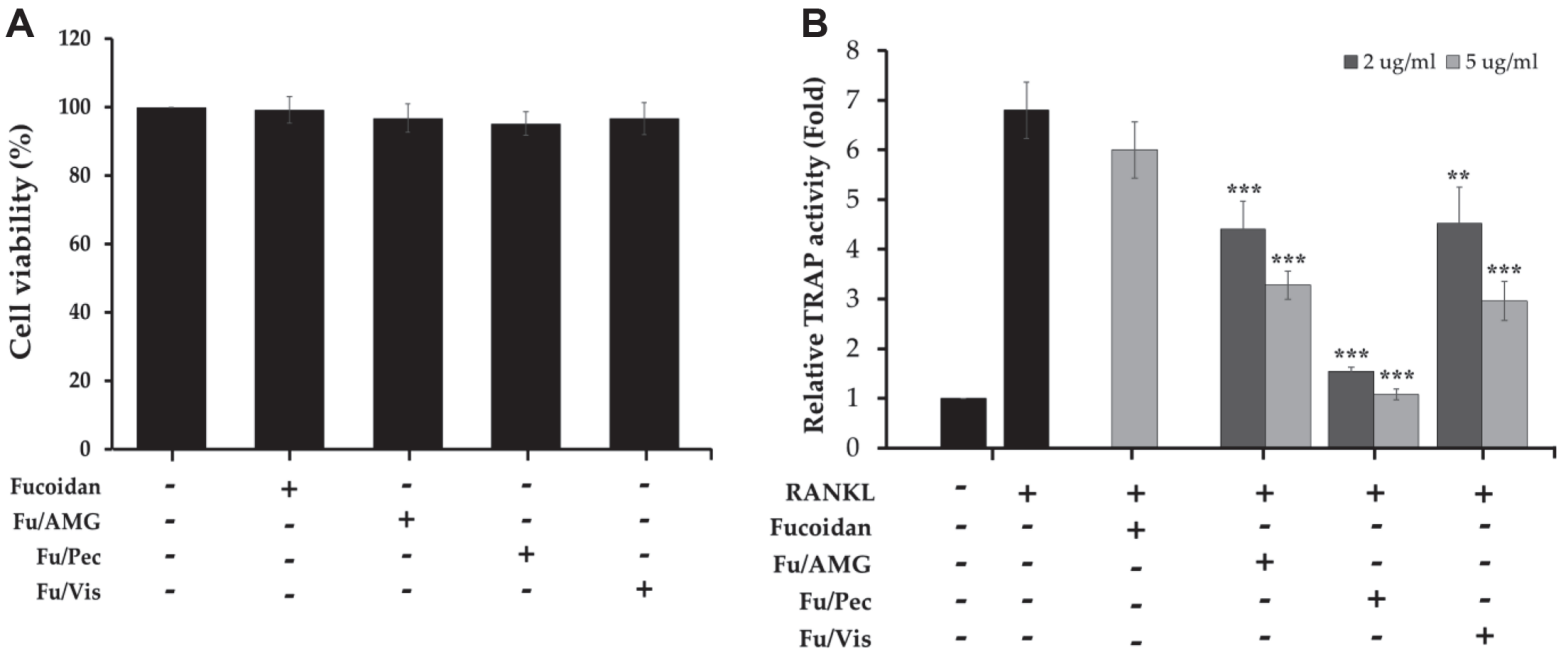
Effect of biotransformed-fucoidan on cell viability and osteoclasts differentiation. (**A**) Cell viabilities of osteoclasts; (**B**) TRAP activities of osteoclasts. Fucoidan was enzymatically biotransformed by hydrolysis with 5% (v/v) of three commercial enzymes, AMG, Pectinex, or Viscozyme, for 2 days at 55°C. Bone marrow-derived macrophages (BMMs) were cultured for 2 days in the presence of M-CSF and then further treated with M-CSF and RANKL, along with 2 μg/ml of biotransformed-fucoidan for 3 days. The cytotoxicities of biotransformed-fucoidans were determined using an MTT assay. To determine TRAP activities, differentiated osteoclasts were stimulated with RANKL in the presence of M-CSF and treated with 2 or 5 μg/ml of biotransformed-fucoidans for 3 days. After cell lysates were treated with TRAP substrate solution, TRAP activities were determined by measuring optical densities of reaction mixtures at 405 nm. The data represented the mean value±SEM of analyses performed in triplicate. **p < 0.01, ***p < 0.001 vs. RANKL only and non-treated group. Fu/AMG: fucoidan hydrolyzed with AMG, Fu/Pec: fucoidan hydrolyzed with Pectinex, Fu/Vis: fucoidan hydrolyzed with Viscozyme.

To examine the cytotoxicity of fucoidans biotransformed with three polysaccharide-hydrolyzing enzymes, an MTT assay was used. Bone marrow-derived macrophages (BMMs) were treated with each biotransformed-fucoidan and incubated for 3 days in the presence of M-CSF and RANKL. As a result, there was not shown any cytotoxicity at 2 μg/ml of each biotransformed-fucoidan on differentiated osteoclasts from BMMs (Fig. 1A). Tartrate-resistant acid phosphatase (TRAP) is highly expressed in chondroclasts and disrupts endochondral bone formation. Actually, TRAP is a differentiation marker of osteoclasts and its regulation might be important for bone homeostasis. After BMMs were cultured with M-CSF and RANKL in the presence of each biotransformed-fucoidan at concentrations of 2 or 5 μg/ml for 3 days, respectively, their TRAP activities were measured as an indicator of osteoclastogenesis. As shown in Fig. 1B, all low-molecularized, biotransformed-fucoidans dose-dependently inhibited RANKL-induced TRAP activity in differentiated osteoclasts from BMMs, even though intact fucoidan had no significant effect at the same doses. Interestingly, biotransformed-fucoidan by Pectinex (Fu/Pec) showed remarkably strongest inhibition against RANKL-induced TRAP activity. It was suggested that biotransformed-fucoidan by Pectinex is considered to regulate the osteoclastogenesis through inhibition of TRAP activity important in the differentiation of osteoclasts and eventually could improve bone homeostasis.

### Ultrafiltration of Fucoidan Biotransformed by Pectinex

**Fig. 2:**
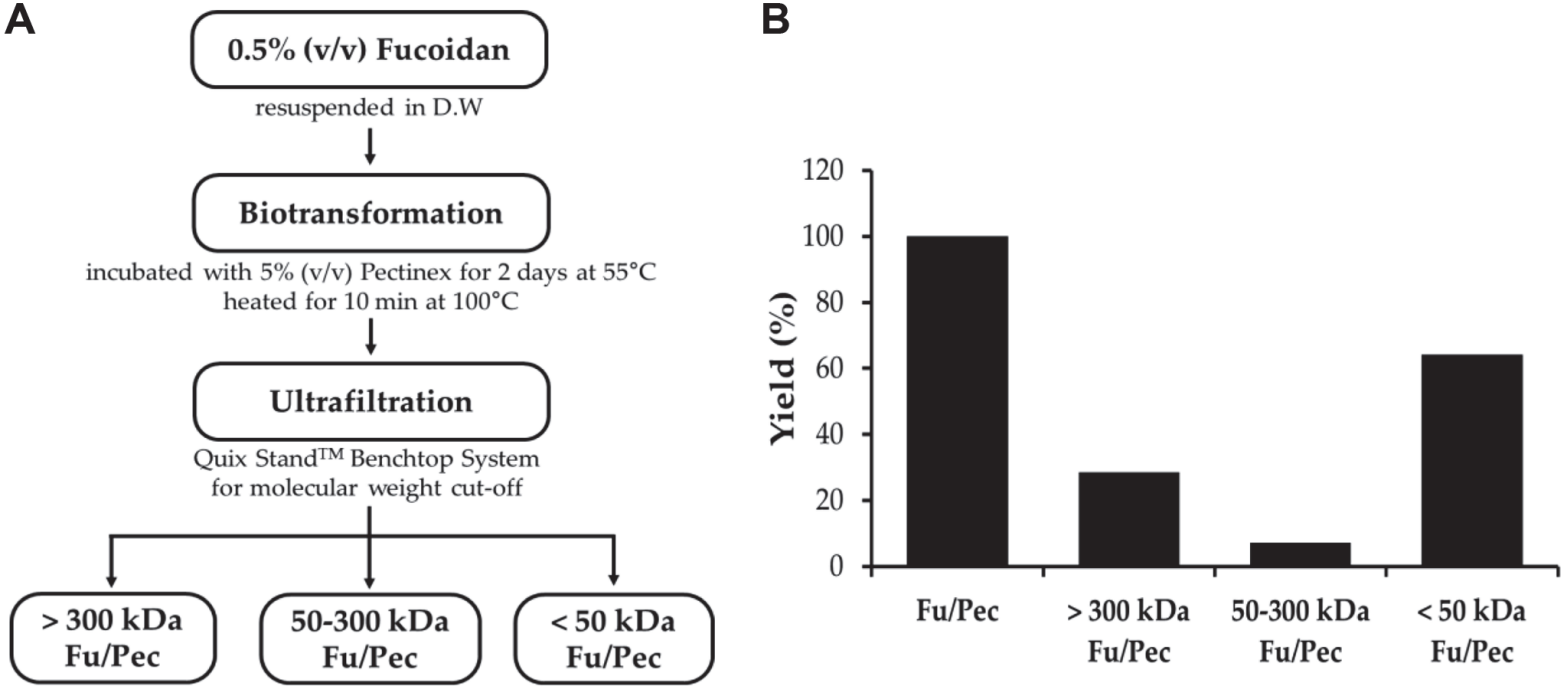
Fractionation of biotransformed-fucoidan by Pectinex (Fu/Pec) by ultrafiltration. (**A**) Schematic process for separation of Fu/Pec fractions; (**B**) Yields of ultrafiltrated fractions. The hydrolysis of fucoidan was carried out as follows. 0.5% of fucoidan was resuspended in distilled water and treated with 5% (v/v) of Pectinex for 2 days at 55°C. The reaction was then stopped by heating for 10 min at 100°C. QuixStand BenchTop Ultrafiltration system was applied to separate >300 kDa, 50- 300 kDa, and <50 kDa Fu/Pec fractions. Fu/Pec: fucoidan hydrolyzed with Pectinex.

Of fucoidan fractions hydrolyzed by several enzymes, biotransformed-fucoidan by Pectinex (Fu/Pec) showed the highest inhibition of TRAP activity in differentiated osteoclast cells from BMMs (Fig. 1B). Fu/Pec was prepared on a larger scale to identify and separate active low-molecular weight of fucoidan fraction using the QuixStandTM BenchTop Ultrafitration System. Fucoidan solution was hydrolyzed with Pectinex at 55°C for 2 days and isolate active fraction from Fu/Pec preparation. Three different molecular weights of fractions were separated to obtain >300 kDa, 50-300 kDa, and <50 kDa MWCO of Fu/Pec (Fig. 2A) at yields of 1,000, 250, and 2,250 mg from total 3,500 mg of biotransformed-fucoidan solution, showing recovery efficiency of roughly 28%, 7%, and 64%, respectively (Fig. 2B).

### Inhibitory Effects of Ultrafiltrated Fractions of Fucoidan Biotransformed by Pectinex on Differentiation of Osteoclast Cells from BMMs

**Fig. 3:**
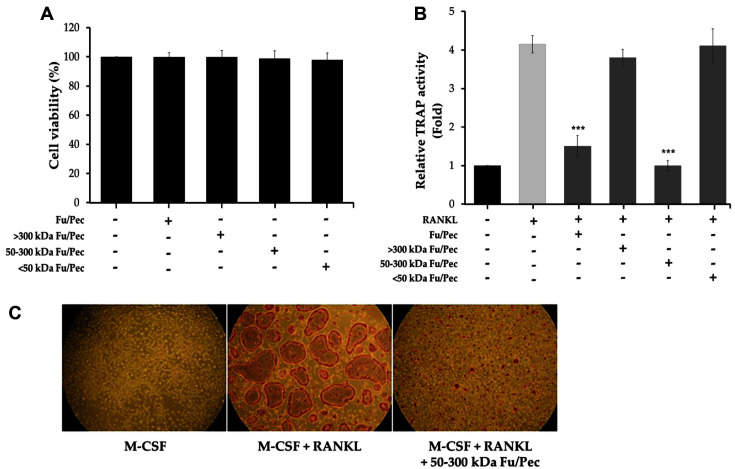
Inhibitory effects of ultrafiltrated Fu/Pec fractions on osteoclast differentiation. (**A**) Cell viabilities of osteoclasts; (**B**) TRAP activities of osteoclasts; (**C**) Detection of TRAP-positive multinucleated cells. Bone marrow-derived macrophages (BMMs) were cultured for 2 days in the presence of M-CSF and then further treated with M-CSF and RANKL for 3 days to differentiate osteoclasts. Cytotoxicities were determined using an MTT assay. To measure TRAP activity, differentiated osteoclasts were cultured with 2 μg/ml of three ultrafiltrated Fu/Pec fractions in the presence of RANKL for 3 days. Cells were then treated with TRAP substrate solution and TRAP activities were determined by measuring optical densities at 405 nm. To observe TRAP-positive multinucleated cells with more than 3 of the number of nuclei, differentiated osteoclasts prepared with same method for TRAP activity were stained using a commercial TRAP staining kit. Cells were photographed under a Leica DM inverted microscope system (Wetzlar, Germany). The data represented the mean value±SEM of analyses performed in triplicate. ***p < 0.001 vs. RANKL only and non-treated group. Fu/Pec: fucoidan hydrolyzed with Pectinex.

It was determined whether some ultrafiltrated fraction from low-molecularized, biotransformed-fucoidan by Pectinex (Fu/Pec) shows an inhibitory effect on osteoclastogenesis. Osteoclast cells differentiated from BMMs, which were stimulated with RANKL in the presence of M-CSF, were treated with each ultrafiltrated fucoidan at a concentration of 2 μg/ml for 3 days. No evidence of cytotoxicities was observed (Fig. 3A). Meanwhile, 50-300 kDa MWCO of Fu/Pec remarkably inhibited RANKL-induced TRAP activity at 2 μg/ml in differentiated osteoclast cells (Fig. 3B), whereas higher and lower molecular weight of Fu/Pec did not show any significant inhibitory effect at the same concentration. To visualize the inhibitory effect of 50-300 kDa Fu/Pec on TRAP induction, RANKL and M-CSF-induced and differentiated osteoclast cells from BMMS were treated with RANKL or/and 2 μg/ml of 50-300 kDa Fu/Pec for 3 days. The cells were stained with TRAP substrate and then photographed under Leica DM inverted microscope system. As a result, RANKL alone enhanced the robust formation of TRAP-positive multinucleated cells but combinatory treatment with RANKL and 50-300 kDa Fu/Pec remarkably reduced the number of TRAP-positive multinucleated cells (Fig. 3C), which was consistent with its inhibition of TRAP enzymatic activity. Taken together, these results suggested that, in particular, 50-300 kDa Fu/Pec has the inhibitory potential on osteoclast differentiation from BMMS by reducing TRAP activity and that lower molecular weight of Fu/Pec remarkably reduces osteoclastogenesis, supposed to be its appropriate molecular weight of fucoidan for higher effectiveness.

### Synergistically Enhanced Effects of Ultrafiltrated Fractions of Fucoidan Biotransformed by Pectinex on Differentiation of MC3T3-E1 cells

**Fig. 4:**
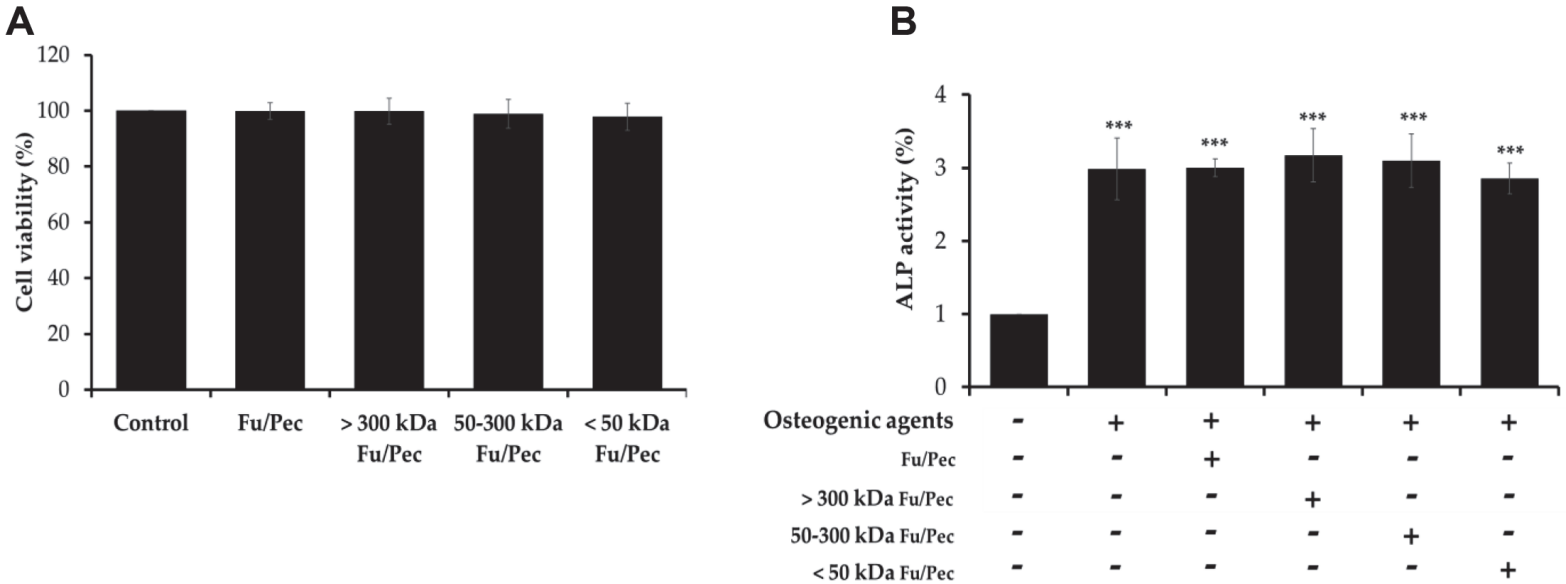
Synergistically enhanced effects of ultrafiltrated Fu/Pec fractions on osteoblast differentiation. (**A**) Cell viabilities of osteoblasts; (**B**) ALP activities of osteoblasts. MC3T3-E1 osteoblastic cells were seeded into a 96-well plate. After 24 h, MC3T3-E1 cells were treated with 2 μg/ml of three ultrafiltrated Fu/Pec fractions for 2 days. Cytotoxicities were determined using an MTT assay. To measure ALP activity, culture media were replaced with fresh α–MEM supplemented with 25 μg/ml of ascorbic acid and 10 mM of β-glycerophosphate as osteogenic agents and cells were then treated with 2 μg/ml of three ultrafiltrated Fu/Pec fractions for 7 days. Cells were then washed and lysed with 100 μl of 0.1% Triton X-100. ALP was analyzed using a reaction mixture containing 2.5 mM *p*-nitrophenyl phosphate (pNPP) solution. Absorbances were measured at 405 nm using a SpectraMax M2e microplate reader (Molecular Devices). The data represented the mean value±SEM of analyses performed in triplicate. ***p < 0.001 vs. non-treated group. Fu/Pec: fucoidan hydrolyzed with Pectinex.

It has been reported that fucoidan itself induces differentiation of MC3T3-E1 osteoblastic cells. So, it was determined whether ultrafiltrated fractions of Fu/Pec have synergistically stimulatory or enhanced effects on osteoblastogenesis. Alkaline phosphatase (ALP) is highly expressed in mineralized tissue cells and is considered a major factor of osteoblast differentiation. To observe the osteoblasts differentiation, MC3T3-E1 cells were cultured in α-MEM containing osteogenic agents supplemented with 25 μg/ml of ascorbic acid and 10 mM of β-glycerophosphate and simultaneously treated with each ultrafiltrated fractions of Fu/Pec at a concentration of 2 μg/ml for 7 days. As a result, there were not observed any cytotoxic effects of these fractions from Fu/Pec on MC3T3-E1 osteoblastic cells (Fig. 4A). Also all ultrafiltrated fractions of Fu/Pec did not has any synergistically encouraging effect on ALP activity of differentiated osteoblast cells stimulated by osteogenic cocktail (Fig. 4B). These results suggested that although 50-300 kDa Fu/Pec might exhibit an inhibitory effect specifically on osteoclastogenesis without synergistically enhancing differentiation of osteoblast cells during osteogenesis.

### Regulatory Effect of 50-300 kDa Fu/Pec on the Expressions of Genes Involved in Differentiation of Osteoclast Cells from BMMs

**Fig. 5:**
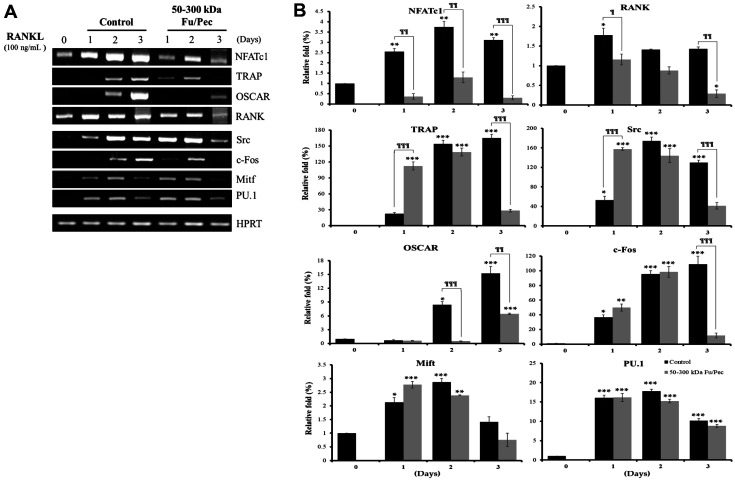
Regulatory effect of 50-300 kDa Fu/Pec on gene expressions involved in differentiation of osteoclast cells. (**A**) Signaling gene expressions in osteoclasts. (**B**) Quantified gene expressions in osteoclasts. Bone marrow-derived macrophages (BMMs) were cultured for 2 days in the presence of M-CSF and then further treated with M-CSF and RANKL for 3 days to differentiate osteoclasts. And differentiated osteoclasts were then cultured with 2 μg/ml of three ultrafiltrated Fu/Pec fractions in the presence of RANKL for 3 days. To assess the expression of indicated genes, total RNA was extracted with Trizol reagent at indicated time point and analyzed by RT-PCR. The results were representative of at least two independent experiments. The data represented the mean value±SEM of analyses performed in duplicate. *p < 0.05, **p < 0.01 and ***p < 0.001 vs. no RANKL and non-treated group. ^¶^*p* < 0.05, ^¶¶^*p* < 0.01 and ^¶¶¶^*p* < 0.001 vs. RANKL and non-treated group. Control: non-treated, Fu/Pec: fucoidan hydrolyzed with Pectinex.

To further determine the inhibitory effect of 50-300 kDa Fu/Pec, the relevant gene expressions were determined by reverse transcription polymerase chain reaction (RT-PCR) after 50-300 kDa Fu/Pec treatment. RANKL binds to RANK, increases the expression of NFATc1, and finally induces TRAP and OSCAR, which are required for the differentiation of osteoclast from BMMs during osteoclastogenesis. Osteoclast cells differentiated from BMMs with M-CSF and RANKL were treated with 2 μg/ml of 50-300 kDa Fu/Pec for 3 days. As was expected, levels of NFATc1, TRAP, OSCAR, and RANK, which are osteoclast-specific differentiation factors, were dramatically upregulated up to 3 days of incubation in the presence of RANKL, whereas 50-300 kDa Fu/Pec significantly inhibited these RANKL-induced gene expressions (Figs. 5A and 5B, upper), which were consistent with the observation that 50-300 kDa Fu/Pec inhibited TRAP activity and TARP-positive multinucleated cell numbers. In addition, expression patterns of Src, c-Fos, and Mitf, which are activated by different differentiation stages, were similar patterns to those of NFATc1, TRAP, OSCAR, and RANK, but not that of PU.1 (Figs. 5A and 5B, lower). Taken together, these results indicated that 50-300 kDa Fu/Pec downregulated the osteoclastogenic signaling cascade of relevant transcription factors acting at each stage and thus inhibited TRAP activity, essential for differentiation of osteoclast cells.

## Discussion

Osteoporosis is a bone-related disease caused by an imbalance of homeostasis between osteoblasts and osteoclasts and thus, the current main strategies involve enhancing osteogenesis or suppressing osteoclastogenesis. Fucoidan, a polysaccharide found in brown seaweeds, is gradually being recognized as a supplement that promotes bone health [15-20]. It has been reported that low-molecular weight of fucoidan inhibits the differentiation of osteoclasts and reduces osteoporosis in ovariectomized rats [17]. Enzymatic hydrolysis, also called biotransformation, is a simple and effective process, whereby a cocktail of microbial-derived enzymes or microorganisms cut cleavages of moleculr bonds to transform complex substances into usable simple materials. Several commercial enzymes are used for enzyme-assisted extraction to enhance the biological activities of extracts [35-37]. In the present study, lower molecular weight of fucoidan produced using polysaccharide hydrolase and ultrafiltration was evaluated for its enhanced inhibitory effect on osteoclast differentiation.

Of the used carbohydrolases, biotransformed-fucoidan by Pectinex (Fu/Pec) significantly strongest inhibited tartrate-resistant acid phosphatase (TRAP) activity during osteoclast differentiation (Fig. 1). Probably Pectinex was suitable to highly depolymerize fucoidan, cell-wall polysaccharide of macroalgae, yielding low-molecularized fucoidans with enhanced anti-osteoclastogenesis activity. Fu/Pec preparation was fractionated into three different sizes of fucoidan by ultrafiltration, yielding as >300 kDa, 50-300 kDa, and <50 kDa MWCO of Fu/Pec (Fig. 2B). Treatment with 50-300 kDa Fu/Pec significantly attenuated RANKL-induced TRAP activity (Fig. 3B) and reduced RANKL-induced TRAP-positive multinucleated osteoclast cell numbers (Fig. 3C). It was supposed that greater TRAP inhibition shown by 50-300 kDa Fu/Pec may have been due to exposure of the optimal sulfate group of fucoidan generated by Pectinex treatment, because previous reports have attributed the efficacy of fucoidan to its residues and the position of the sulfated region [22, 38-40]. Taken together, our results suggested that higher anti-osteoclastogenic activity of 50-300 kDa Fu/Pec is due to its relevant molecular size and the presence of the sulfated groups, reduced from intact fucoidan structure.

Fucoidan has been reported to enhance osteoblast growth and differentiation via several pathways. In addition, fucoidan strongly induces osteogenesis-specific markers such as alkaline phosphatase (ALP), osteopontin, type I collagen, Runx2 and osteocalcin in human adipose-derived stem cells and positively upregulates BMP2 signaling, which is essential for osteogenesis, through JNK- and ERK-dependent BMP2-Smad signaling [7, 8, 16]. Ultrafiltrated fractions of Fu/Pec were examined to determine whether they synergistically enhance osteogenesis of MC3T3-E1 osteoblastic cells stimulated with osteogenic agents. Unlike expected, none of Fu/Pec fractions promoted osteoblast differentiation (Fig. 4). Thus, our results suggested that 50-300 kDa Fu/Pec strongly inhibits osteoclastogenesis without any significant impact on boosting osteogenesis.

Osteoclasts are derived from hematopoietic precursors and partly from differentiations of monocytes/macrophages. Hematopoietic precursors secrete cytokines such as the macrophage stimulating factor (M-CSF) and receptor for activation of nuclear factor-κB ligand (RANKL). RANKL is known to induce the expressions and activities of transcription factors such as Mi transcription factor (Mitf), PU.1, and nuclear factor of activated T cells c1 (NFATc1) to promote the differentiation of osteoclasts [4-6]. M-CSF and RANKL induce the formation of TRAP-positive multinuclear osteoclasts from bone marrow-derived macrophages (BMMs). Upon stimulation, activated MAP kinases regulate transcription factors such as Mitf and NFATc1, subsequently leading to the upregulation of the osteoclast-associated receptor (OSCAR) gene. This study demonstrated that 50-300 kDa Fu/Pec remarkably suppressed the gene expressions of NFATc1, TRAP, OSCAR, RANK, Src, c-Fos and Mitf, but not that of PU.1, after culture of BMMs in the presence M-CSF and RANKL for 3 days (Fig. 5). The transcription factor PU.1 participates in osteoblast differentiation and also induce secretion of RANKL to accelerate osteoclastogenesis through successively increasing the expression of their target genes [41]. It could be speculated that inhibition of PU.1 expression seemed to be blunt in osteoclastogenesis, even if its signals were extremely supressed by 50-300 kDa Fu/Pec. Finally, it was found that 50-300 kDa Fu/Pec inhibited differentiation of osteoclast, by suppressing signaling gene expressions at each stage of osteoclast differentiation.

In conclusion, we found fucoidan biotransformed by Petinex potently inhibited TRAP, which is essential for osteoclast differentiation. Furthermore, 50-300 kDa Fu/Pec inhibited osteoclastogenesis by inhibiting TRAP in differentiated osteoclast cells from BMMs, probably due to suitable molecular weight and sulfated groups of biotransformed fucoidan for molecular docking with targets of osteoclastogenesis. This was in line with our RT-PCR results, which showed 50-300 KDa Fu/Pec inhibited the gene expressions of the transcription factors (NFATc1, TRAP, OSCAR, RANK, Src, c-Fos, and Mitf) involved in signaling of osteoclast differentiation. The observation that 50-300 kDa Fu/Pec strongly inhibited osteoclast differentiation suggested its potential use as the treatment of osteoporosis. Further physiological studies and toxicity tests are required to examine its effect of 50-300 kDa Fu/Pec in animal models.

## References

[1] Confavreux CB (2011). Bone: from a reservoir of minerals to a regulator of energy metabolism. Kidney Int..

[2] Al-Bari AA, Al Mamun AA (2020). Current advances in regulation of bone homeostasis. FASEB Bioadv..

[3] Kim JM, Lin C, Stavre Z, Greenblatt MB, Shim JH (2020). Osteoblast-osteoclast communication and bone homeostasis. Cells.

[4] Kim JH, Kim N (2016). Signaling pathways in osteoclast differentiation. Chonnam Med. J..

[5] Takayanagi H, Kim S, Koga T, Nishina H, Isshiki M, Yoshida H (2002). Induction and activation of the transcription factor NFATc1 (NFAT2) integrate RANKL signaling in terminal differentiation of osteoclasts. Dev. Cell.

[6] Pennanen P, Kallionpaa RA, Peltonen S, Nissinen L, Kahari VM, Heerva E (2021). Signaling pathways in human osteoclasts differentiation: ERK1/2 as a key player. Mol. Biol. Rep..

[7] Phimphilai M, Zhao Z, Boules H, Roca H, Franceschi RT (2006). BMP signaling is required for RUNX2-dependent induction of the osteoblast phenotype. J. Bone Miner. Res..

[8] Amarasekara DS, Kim S, Rho J (2021). Regulation of osteoblast differentiation by cytokine networks. Int. J. Mol. Sci..

[9] Golub EE, Boesze-Battaglia K (2007). The role of alkaline phosphatase in mineralization. Curr. Opin. Orthop..

[10] Lotz EM, Lohmann CH, Boyan BD, Schwartz Z (2020). Bisphosphonates inhibit surface-mediated osteogenesis. J. Biomed. Mater. Res. A.

[11] Levin VA, Jiang X, Kagan R (2018). Estrogen therapy for osteoporosis in the modern era. Osteoporos. Int..

[12] Oh S, Ahn SC (2015). Current medical therapies for osteoporosis and its alternative treatments using natural products. Kor. Life Sci..

[13] Zheng X, Lee SK, Chun OK (2016). Soy Isoflavones and osteoporotic bone loss: a review with an emphasis on modulation of bone remodeling. J. Med. Food.

[14] Martiniakova M, Babikova M, Omelka R (2020). Pharmacological agents and natural compounds: available treatments for osteoporosis. J. Physiol. Pharmacol..

[15] Kim YW, Baek SH, Lee SH, Kim TH, Kim SY (2014). Fucoidan, a sulfated polysaccharide, inhibits osteoclast differentiation and function by modulating RANKL signaling. Int. J. Mol. Sci..

[16] Kim BS, Kang HJ, Park JY, Lee J (2015). Fucoidan promotes osteoblast differentiation via JNK- and ERK-dependent BMP2-Smad 1/5/8 signaling in human mesenchymal stem cells. Exp. Mol. Med..

[17] Jin X, Zhu L, Li X, Jia J, Zhang Y, Sun X (2017). Low-molecular weight fucoidan inhibits the differentiation of osteoclasts and reduces osteoporosis in ovariectomized rats. Mol. Med. Rep..

[18] Lu SH, Hsia YJ, Shih KC, Chou TC (2019). Fucoidan prevents RANKL-stimulated osteoclastogenesis and LPS-induced inflammatory bone loss via regulation of Akt/GSK3beta/PTEN/NFATc1 signaling pathway and calcineurin activity. Mar. Drugs.

[19] Park SJ, Lee KW, Lim DS, Lee S (2011). The sulfated polysaccharide fucoidan stimulates osteogenic differentiation of human adiposederived stem cells. Stem Cells Dev..

[20] Cho YS, Jung WK, Kim JA, Choi IW, Kim SK (2009). Beneficial effects of fucoidan on osteoblastic MG-63 cell differentiation. Food Chem..

[21] Lee YK, Lim DJ, Lee YH, Park YI (2006). Variation in fucoidan contents and monosaccharide compositions of Korean *Undaria pinnatifida* (Harvey) Suringar (Phaeophyta). Algae.

[22] Zayed A, El-Aasr M, Ibrahim AS, Ulber R (2020). Fucoidan characterization: determination of purity and physicochemical and chemical properties. Mar. Drugs.

[23] van Weelden G, Bobinski M, Okla K, van Weelden WJ, Romano A, Pijnenborg JMA (2019). Fucoidan structure and activity in relation to anti-cancer mechanisms. Mar. Drugs.

[24] Mansour MB, Balti R, Yacoub L, Ollivier V, Chaubet F, Maaroufi RM (2019). Primary structure and anticoagulant activity of fucoidan from the sea cucumber *Holothuria polii*. Int. J. Biol. Macromol..

[25] Min SK, Kwon OC, Lee S, Park KH, Kim JK (2012). An antithrombotic fucoidan, unlike heparin, does not prolong bleeding time in a murine arterial thrombosis model: a comparative study of *Undaria pinnatifida* sporophylls and *Fucus vesiculosus*. Phytother. Res..

[26] Apostolov E, Lukova P, Baldzhieva A, Katsarov P, Nikolova M, Iliev I (2020). Immunomodulatory and anti-inflammatory effects of fucoidan: a review. Polymers (Basel).

[27] Wang W, Wu J, Zhang X, Hao C, Zhao X, Jiao G (2017). Inhibition of influenza a virus infection by fucoidan targeting viral neuraminidase and cellular EGFR pathway. Sci. Rep..

[28] Wang Y, Xing M, Cao Q, Ji A, Liang H, Song S (2019). Biological activities of fucoidan and the factors mediating its therapeutic effects: a review of recent studies. Mar. Drugs 2019.

[29] Yang JY, Lim SY (2019). Fucoidans and bowel health. Mar. Drugs.

[30] Wang X, Shan X, Dun Y, Cai C, Hao J, Li G (2019). Anti-metabolic syndrome effects of fucoidan from Fucus vesiculosus via reactive oxygen species-mediated regulation of JNK, Akt, and AMPK signaling. Molecules.

[31] Wu SY, Yang WY, Cheng CC, Lin KH, Sampurna BP, Chan SM (2020). Low molecular weight fucoidan inhibits hepatocarcinogenesis and nonalcoholic fatty liver disease in zebrafish via ASGR/STAT3/HNF4A signaling. Clin. Transl. Med..

[32] Wang J, Zhang Q, Zhang Z, Song H, Li P (2010). Potential antioxidant and anticoagulant capacity of low molecular weight fucoidan fractions extracted from *Laminaria japonica*. Int. J. Biol. Macromol..

[33] Zhao X, Guo F, Hu J, Zhang L, Xue C, Zhang Z (2016). Antithrombotic activity of oral administered low molecular weight fucoidan from *Laminaria japonica*. Thromb. Res..

[34] Cui W, Zheng Y, Zhang Q, Wang J, Wang L, Yang W (2014). Low-molecular-weight fucoidan protects endothelial function and ameliorates basal hypertension in diabetic Goto-Kakizaki rats. Lab. Invest..

[35] Madeira JV, Teixeira CB, Macedo GA (2015). Biotransformation and bioconversion of phenolic compounds obtainment: an overview. Crit. Rev. Biotechnol..

[36] Singh R (2017). Microbial biotransformation: a process for chemical alterations. J. Bacteriol. Mycol..

[37] Kee SH, Chiongson JBV, Saludes JP, Vigneswari S, Ramakrishna S, Bhubalan K (2021). Bioconversion of agro-industry sourced biowaste into biomaterials via microbial factories - A viable domain of circular economy. Environ. Pollut..

[38] Li B, Lu F, Wei X, Zhao R (2008). Fucoidan: structure and bioactivity. Molecules.

[39] Morya VK, Kim J, Kim EK (2012). Algal fucoidan: structural and size-dependent bioactivities and their perspectives. Appl. Microbiol. Biotechnol..

[40] Ale MT, Mikkelsen JD, Meyer AS (2011). Important determinants for fucoidan bioactivity: a critical review of structure-function relations and extraction methods for fucose-containing sulfated polysaccharides from brown seaweeds. Mar. Drugs.

[41] So H, Rho J, Jeong D, Park R, Fisher DE, Ostrowski MC (2003). Microphthalmia transcription factor and PU.1 synergistically induce the leukocyte receptor osteoclast-associated receptor gene expression. J. Biolog. Chem..

[42] Rethineswaran VK, Kim YJ, Jang WB, Ji ST, Kang S, Kim DY (2019). Enzyme-aided extraction of fucoidan by AMG augments the functionality of EPCs through regulation of the AKT/Rheb signaling pathway. Mar. Drugs.

[43] Lee JW, Kim JH, Kim K, Jin HM, Lee KB, Chung DJ (2007). Ribavirin enhances osteoclast formation through osteoblasts via upregulation of TRANCE/RANKL. Mol. Cell. Biochem..

